# Cerebral Autosomal Dominant Arteriopathy With Subcortical Infarcts and Leukoencephalopathy (CADASIL) in a 32-Year-Old Male Presenting With a Transient Ischemic Attack (TIA)

**DOI:** 10.7759/cureus.70970

**Published:** 2024-10-06

**Authors:** Rabail Karim, Munzir Malik, Hamza Cheema, Abdul Aziz, Rimsha Khan

**Affiliations:** 1 Neurology, The Walton Centre, Liverpool, GBR; 2 Acute Medicine, Wrexham Maelor Hospital, Wrexham, GBR

**Keywords:** and transient ischemic attack (tia), cerebral autosomal dominant arteriopathy with subcortical infarcts and leukoencephalopathy (cadasil), cerebro-vascular accident (stroke), migraine disorder, vascular spasm

## Abstract

This case report describes a 32-year-old male with a familial history of cerebral autosomal dominant arteriopathy with subcortical infarcts and leukoencephalopathy (CADASIL), presenting with right-sided weakness and visual disturbances. The symptoms, consistent with a transient ischemic attack (TIA), resolved spontaneously. Subsequent evaluation, including MRI brain imaging, revealed a high T2 signal in subcortical white matter in the frontal and parietal lobes, consistent with CADASIL. Positive NOTCH3 testing confirmed the diagnosis, leading to the initiation of antiplatelet and statin therapy under the care of both stroke and neurology teams.

## Introduction

Cerebral autosomal dominant arteriopathy with subcortical infarcts and leukoencephalopathy (CADASIL) is a rare hereditary small vessel disease characterized by progressive vascular pathology leading to recurrent ischemic events in the brain [1]. First identified in the 1990s, CADASIL has since emerged as a distinctive entity with a genetic basis, primarily associated with mutations in the NOTCH3 gene located on chromosome 19.

The hallmark of CADASIL lies in the dysfunction of vascular smooth muscle cells within small arteries, leading to the deposition of granular osmiophilic material (GOM) in vessel walls [1,2]. This pathological process results in subcortical infarcts, white matter changes, and cognitive decline, often presenting challenges in diagnosis due to its varied clinical manifestations.

While the typical age of onset is in the fourth or fifth decade, this case report elucidates a noteworthy instance of CADASIL manifesting in a 32-year-old male. The patient's unique clinical presentation, characterized by right-sided weakness and visual disturbances, prompted a comprehensive diagnostic evaluation, ultimately revealing characteristic radiological findings consistent with CADASIL.

Moreover, the familial history of CADASIL in the patient's mother, coupled with a background of migraines, underscores the hereditary nature of the disorder and the importance of recognizing potential genetic predispositions in at-risk individuals. This case sheds light on the intricate interplay between genetic factors, clinical presentation, and diagnostic modalities in the identification and management of CADASIL.

In this context, the discussion will delve into the patient's clinical course, the significance of neuroimaging in confirming the diagnosis, the genetic confirmation through NOTCH3 testing, and the multidisciplinary approach involving stroke and neurology teams for effective management [2]. The complexities surrounding CADASIL, including challenges in diagnosis and the need for familial screening, highlight the necessity for a holistic understanding of this condition to optimize patient care and outcomes.

## Case presentation

A 32-year-old male, whose mother had a confirmed diagnosis of CADASIL with a history of migraines, presented with right-sided weakness and visual disturbances.

There is limited medical history but a significant family history of CADASIL in the patient's mother, who also had a history of migraines. Upon examination, the patient demonstrated no residual weakness, and his neurological examination was otherwise unremarkable. However, given the transient nature of the symptoms, the initial concern was for a possible transient ischemic attack (TIA). The symptoms resolved spontaneously after a few hours, prompting evaluation by the stroke team, who initially treated the episode as a TIA.

Neuroimaging findings

**Figure 1:**
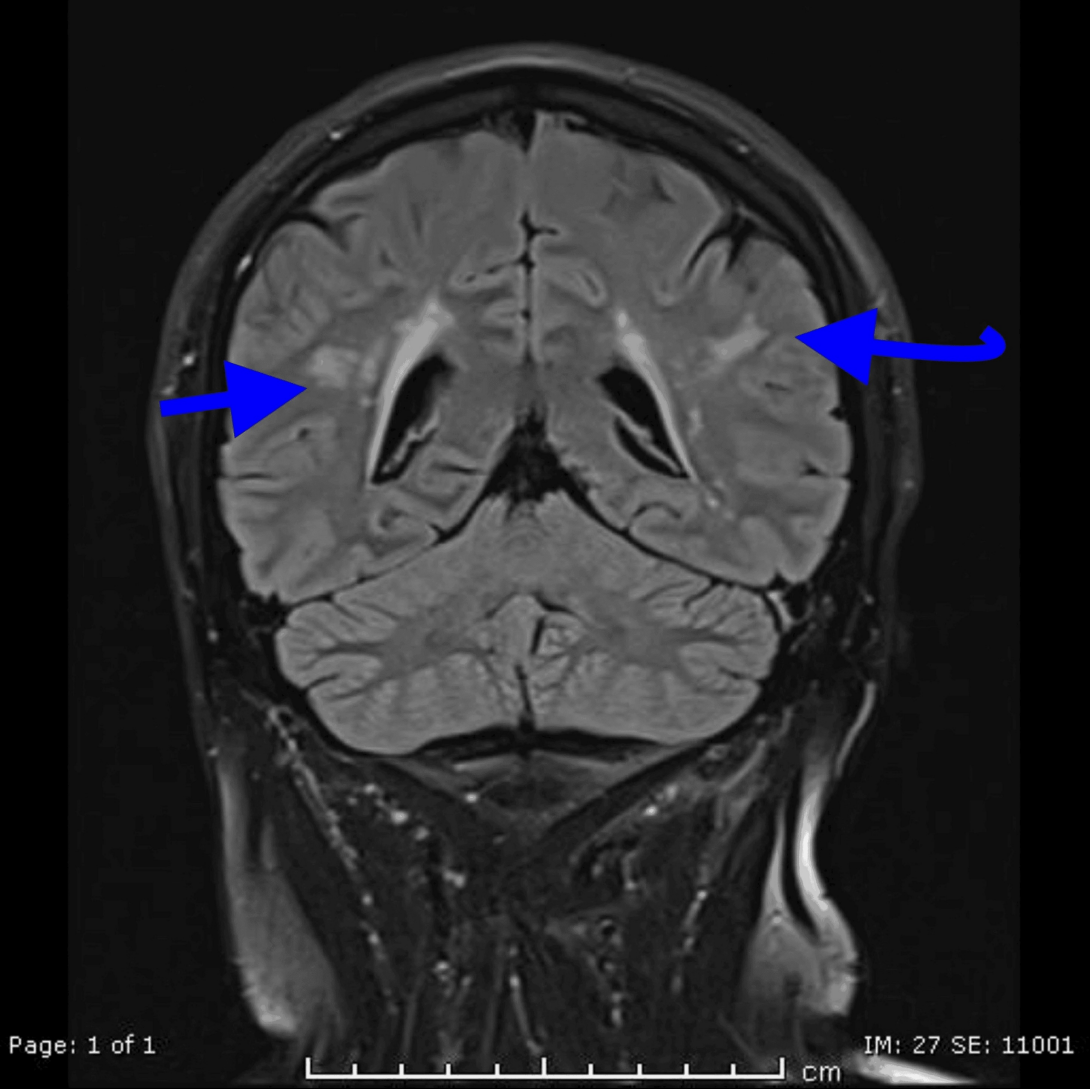
MRI T2 coronal view of the patient shows the presence of a high T2 signal in the subcortical white matter of the frontal and parietal lobes, which is a characteristic radiological feature of CADASIL CADASIL, cerebral autosomal dominant arteriopathy with subcortical infarcts and leukoencephalopathy

**Figure 2:**
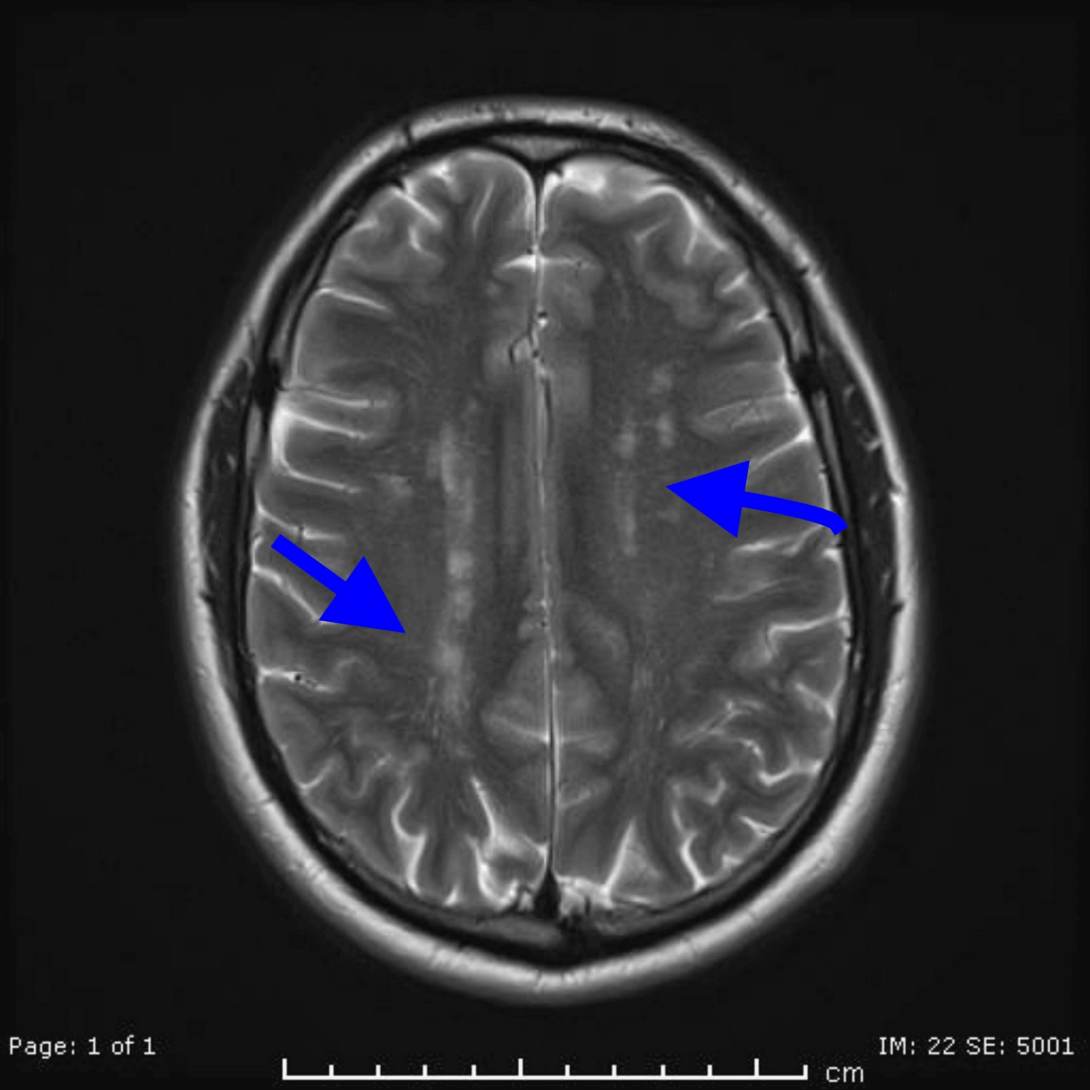
MRI (T2 axial view) of the patient showing findings in keeping with CADASIL CADASIL, cerebral autosomal dominant arteriopathy with subcortical infarcts and leukoencephalopathy

The MRI brain imaging played a pivotal role in establishing the diagnosis. The presence of a high T2 signal in the subcortical white matter of the frontal and parietal lobes showed a characteristic radiological feature of CADASIL (Figure 1 and Figure 2). This finding reflects the ischemic changes in the small vessels of the brain, contributing to the understanding of the disease's pathophysiology [3].

Genetic confirmation

The positive NOTCH3 testing further confirmed the diagnosis of CADASIL. Genetic testing is crucial in cases where clinical and imaging findings are suggestive but not definitive, providing a conclusive diagnosis and allowing for appropriate genetic counseling.

The patient was initiated on antiplatelet therapy and statins, aligning with the current management approach for CADASIL [4]. The goal is to prevent further ischemic events and manage vascular risk factors. A multidisciplinary care approach involving stroke and neurology teams was adopted, which is essential to address both acute and long-term aspects of the disease.

## Discussion

This case report highlights a 32-year-old male presenting with TIA-like symptoms, which led to the eventual diagnosis of CADASIL, confirmed through neuroimaging and genetic testing. His presentation, imaging findings, and family history align closely with what is observed in the existing literature on CADASIL, though certain aspects, particularly the early onset and subtle initial symptoms, offer important insights into the variability of this condition [3].

Early-onset CADASIL and migraine predominance

The patient’s age at presentation is younger than the average age of onset commonly reported for CADASIL, which tends to occur in the fourth or fifth decade of life. Studies suggest that the median age for the first ischemic event in CADASIL patients is typically around 45-50 years [4]. In this case, the patient experienced symptoms of right-sided weakness and visual disturbance in his early 30s, suggesting that the age of onset can be highly variable, particularly in those with a strong family history [4].

Migraines with aura, which were prevalent in this patient’s family, are often an early symptom of CADASIL, preceding ischemic events for years or even decades. In fact, approximately 20-40% of CADASIL patients report migraine as their initial symptom, often starting in their 20s or 30s [5]. The presence of migraines in both the patient and his mother, who had a proven diagnosis of CADASIL, is consistent with existing reports on the hereditary nature of migraine in this condition [6]. It underscores the need for clinicians to consider CADASIL in young adults with migraines, especially if accompanied by transient neurological symptoms.

TIA-like presentation and its challenges

This patient’s presenting symptoms of right-sided weakness and visual disturbances were treated initially as a TIA, which is a common misdiagnosis in CADASIL. CADASIL often presents with recurrent ischemic events that mimic TIA or minor strokes [5]. The self-limiting nature of the patient’s symptoms fits within this pattern but underscores a diagnostic challenge that is noted in the literature: differentiating true TIA or stroke from CADASIL-related vascular episodes. While the resolution of symptoms in a few hours is typical of a TIA, the subsequent MRI findings pointed toward the underlying small vessel disease seen in CADASIL [5,7]. In CADASIL, ischemic events may not always be associated with classic risk factors for cerebrovascular disease, such as hypertension or diabetes, making the diagnosis more challenging in younger individuals without clear vascular risk factors [8]. This reinforces the need for early neuroimaging and genetic testing, especially when there is a suggestive family history.

Neuroimaging in CADASIL

MRI brain findings are critical in diagnosing CADASIL and played a key role in this case. The patient’s MRI showed high T2-weighted signal intensities in the subcortical white matter of the frontal and parietal lobes, which is characteristic of CADASIL [4]. The literature widely recognizes such white matter hyperintensities as a hallmark feature of the disease, often appearing well before clinical symptoms manifest. Lesions in the frontal, temporal, and parietal lobes, as well as in the external capsule and periventricular areas, are frequently described. The presence of such findings, especially in the absence of other risk factors for cerebrovascular disease, is highly suggestive of CADASIL [5,8].

Comparatively, the extent and location of white matter changes in this patient are consistent with the more advanced stages of CADASIL. As seen in other studies, while these findings are generally progressive, they can be present even in younger, asymptomatic individuals who carry the NOTCH3 mutation [5].

Genetic testing and family history

The role of genetic testing in diagnosing CADASIL is well established, with mutations in the NOTCH3 gene being responsible for the disease. In this case, positive NOTCH3 testing confirmed the diagnosis. The hereditary nature of CADASIL is also well documented, with a significant chance of passing the NOTCH3 mutation to offspring in an autosomal dominant pattern [6]. The patient’s family history, including a mother with proven CADASIL, aligns with the inheritance patterns seen in existing literature [7].

The presence of familial CADASIL often prompts earlier screening in at-risk individuals. In this case, the family history and positive genetic testing helped solidify the diagnosis and ensured that appropriate preventive measures were taken.

Management and prognosis

There is no curative treatment for CADASIL, and management primarily focuses on preventing further ischemic events and managing symptoms. The patient was started on antiplatelet therapy and a statin, consistent with common management strategies for reducing the risk of future strokes [8]. Although CADASIL is a small vessel disease, existing guidelines generally recommend antiplatelet therapy for secondary prevention of ischemic events despite the lack of direct evidence specific to CADASIL.

In terms of prognosis, CADASIL is a progressive condition with increasing disability over time due to recurrent strokes, cognitive decline, and, eventually, dementia. This patient’s early diagnosis, made possible through imaging and genetic testing, will allow for closer monitoring and early interventions to mitigate the disease’s progression [9].

This case illustrates the diagnostic and management challenges posed by CADASIL, especially in younger patients. The presentation of TIA-like symptoms, the role of MRI in diagnosis, and the subsequent genetic confirmation through NOTCH3 testing all align with the broader understanding of CADASIL in the literature [10]. It emphasizes the need for heightened clinical awareness, particularly in patients with a familial history of the disease, as early diagnosis and management may improve long-term outcomes.

## Conclusions

In conclusion, this case underscores the complexity of diagnosing CADASIL, emphasizing the significance of clinical acumen, neuroimaging, and genetic testing. Interdisciplinary collaboration and a comprehensive management approach are vital for optimizing outcomes in patients with this rare genetic vascular disorder. This case highlights the intricate interplay between clinical presentation, imaging, and genetic testing in diagnosing CADASIL. The recognition of familial patterns, coupled with a multidisciplinary approach involving stroke and neurology teams, is crucial for both acute management and long-term care in individuals with this rare genetic vascular disorder.

## References

[1] Chitnis T, Hollmann TJ (2012). CADASIL mutation and Balo concentric sclerosis: a link between demyelination and ischemia?. Neurology.

[2] Di Donato I, Bianchi S, De Stefano N (2017). Cerebral Autosomal Dominant Arteriopathy with Subcortical Infarcts and Leukoencephalopathy (CADASIL) as a model of small vessel disease: update on clinical, diagnostic, and management aspects. BMC Med.

[3] Lesnik Oberstein SA, van den Boom R, van Buchem MA (2001). Cerebral microbleeds in CADASIL. Neurology.

[4] Duering M, Righart R, Csanadi E, Jouvent E, Hervé D, Chabriat H, Dichgans M (2012). Incident subcortical infarcts induce focal thinning in connected cortical regions. Neurology.

[5] Auer DP, Pütz B, Gössl C, Elbel G, Gasser T, Dichgans M (2001). Differential lesion patterns in CADASIL and sporadic subcortical arteriosclerotic encephalopathy: MR imaging study with statistical parametric group comparison. Radiology.

[6] Gladstone JP, Dodick DW (2005). Migraine and cerebral white matter lesions: when to suspect cerebral autosomal dominant arteriopathy with subcortical infarcts and leukoencephalopathy (CADASIL). Neurologist.

[7] Rutten JW, Dauwerse HG, Gravesteijn G (2016). Archetypal NOTCH3 mutations frequent in public exome: implications for CADASIL. Ann Clin Transl Neurol.

[8] Joutel A, Corpechot C, Ducros A (1996). Notch3 mutations in CADASIL, a hereditary adult-onset condition causing stroke and dementia. Nature.

[9] Lahkim M, Laamrani FZ, Andour H, Gharbaoui Y, Sanhaji L, El-Fenni J, En-Nouali H (2021). Cadasil syndrome: a case report with a literature review. Radiol Case Rep.

[10] Tournier-Lasserve E, Iba-Zizen MT, Romero N, Bousser MG (1991). Autosomal dominant syndrome with strokelike episodes and leukoencephalopathy. Stroke.

